# Temporal walk based centrality metric for graph streams

**DOI:** 10.1007/s41109-018-0080-5

**Published:** 2018-08-14

**Authors:** Ferenc Béres, Róbert Pálovics, Anna Oláh, András A. Benczúr

**Affiliations:** 1 Institute for Computer Science and Control, Hungarian Academy of Sciences, (MTA SZTAKI), Kende Street 13-17, Budapest, H-1111 Hungary; 2 Eötvös University Budapest, Pázmány s. 1, Budapest, H-1117 Hungary; 3 Department of Computer Science, Stanford University, 353 Serra Mall, Stanford, 94305 CA USA; 4 Max Planck Institute for Informatics, 4 Stuhlsatzenhausweg, Saarbrücken, 66123 Germany

**Keywords:** Temporal graphs, Centrality, Twitter measurement, Dynamics of social networks, Social media analysis: blogs and friendship networks

## Abstract

A plethora of centrality measures or rankings have been proposed to account for the importance of the nodes of a network. In the seminal study of Boldi and Vigna (2014), the comparative evaluation of centrality measures was termed a difficult, arduous task. In networks with fast dynamics, such as the Twitter mention or retweet graphs, predicting emerging centrality is even more challenging.

Our main result is a new, temporal walk based dynamic centrality measure that models temporal information propagation by considering the order of edge creation. Dynamic centrality measures have already started to emerge in publications; however, their empirical evaluation is limited. One of our main contributions is creating a quantitative experiment to assess temporal centrality metrics. In this experiment, our new measure outperforms graph snapshot based static and other recently proposed dynamic centrality measures in assigning the highest time-aware centrality to the actually relevant nodes of the network. Additional experiments over different data sets show that our method perform well for detecting concept drift in the process that generates the graphs.

## Introduction

There is a wide range of commercial and research applications devoted to identifying important, popular, and influential users on social media platforms (Diakopoulos et al. 2012). Since popularity and importance are social phenomena and judged in a social context, a way to quantify them is through a complex combination of social and behavioral factors. These often include graph characteristics like degree, PageRank, and other centrality metrics (Bakshy et al. 2011; Chang et al. 2013; Pal and Counts 2011; Weng et al. 2010) measured over the social network. The definitions of centrality can vary greatly and can incorporate both global and local factors of a user’s location within the social network (Boldi and Vigna 2014).

**Fig. 1:**
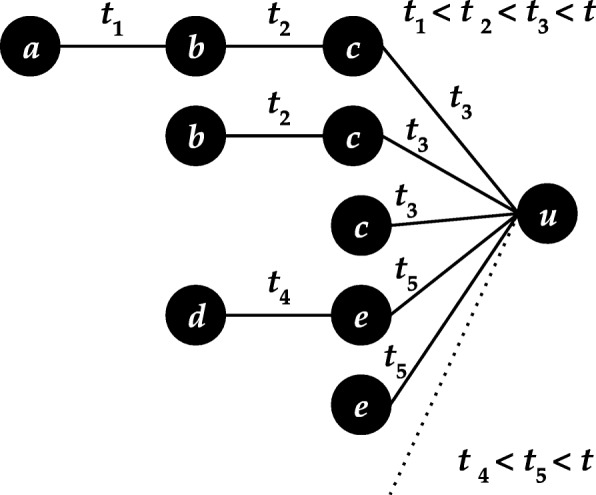
Temporal walks ending at node *u* before time *t*

In this work we present **temporal Katz centrality**, an online updateable graph centrality metric for tracking and measuring user importance over time. We consider temporal networks where the edges of the network arrive continuously in time. In other words the graph is represented as a sequence of time-stamped edges (Rozenshtein and Gionis 2016). Our proposed metric is based on the concept of time-respecting walks containing a sequence of adjacent edges with timestamps ordered in time. As seen in Fig. 1, for node *u* temporal Katz centrality aggregates each temporal walk ending before time *t* at *u*.

Online updateability poses computational restrictions and challenges to most centrality measures and graph algorithms in general. In this paper we consider the data stream model (Babcock et al. 2002). The rationale of the streaming model lies in the size and complexity of real-world networks: If we collect data for the range of hours to process as a graph snapshot, we impose additional delay on the prediction, since processing the entire graph snapshot will be time-consuming. In this sense, our new method can be considered a graph algorithm for online machine learning (Bifet et al. 2010).

Although many studies tried to identify the best estimates for the importance of a social media user, to the best of our knowledge, there are only two previous studies (Rozenshtein and Gionis 2016; Ghanem et al. 2017) that propose **data stream updateable** centrality measures. The algorithm of (Rozenshtein and Gionis 2016), which we analyze in Section Temporal PageRank, cannot incorporate the actual edge arrival times in its calculations. We believe our method is superior in using the exact time of interaction between two social media users, resulting in better performance in our prediction task. The algorithm of (Ghanem et al. 2017) can be best described as a heuristic version of betweenness centrality to “ego-graphs”, which have paths of length two only. They applied their algorithms for small graphs of less than 250 nodes only. Based on the comparative evaluation of centrality measures in (Boldi and Vigna 2014), we chose not to include experiments with betweenness centrality in our experiments.

Another key issue that we address is the difficulty of the timely evaluation of fast changes in social media. In order to evaluate a static centrality measure, static ground truth labeling is required, which itself often requires tedious human effort. In (Boldi and Vigna 2014), for example, the Text Retrieval Conference (TREC) topics are used (Clarke et al. 2004). In a dynamic graph, depending on time granularity, the same human data curation may be required in each time step. For example, in the study most similar to ours (Rozenshtein and Gionis 2016), only small temporal social network snapshots are collected, and evaluation is mostly based on convergence to static centrality measures.

In our best effort to provide quantitative evaluation for dynamic centrality, we consider daily granularity and **compile ground truth** based on an external source. We collect tweets about Roland-Garros 2017, the French Open Tennis Tournament (RG17), and US Open 2017, the United States Open Tennis Tournament (UO17). We compute both static and dynamic centrality metrics over the time-aware mention graph that we extract from the tweets. We define the mention graph by adding a time-stamped edge (*u,v,t*) whenever user *u* mentions *v* in a tweet at time *t*. For ground truth, we consider the Twitter accounts of players participating in daily rounds as relevant. We then hour by hour investigate how mentions of players for the coming day take over the importance of past participants.

In this paper, we design and evaluate an online updateable, dynamic graph centrality measure. Our main contribution is threefold:

- We propose a new, online updateable path count based centrality measure as a temporal variant of the successful Katz index (Katz 1953). Our measure incorporates arbitrary time decay functions that can be adapted to the task in question.
- We compile a data set with ground truth labels for the quantitative evaluation of dynamic centrality. Our evaluation is based on our Twitter collection about tennis tournaments. For centrality ground truth at a given time, we set the players participating in rounds on given days.
- We experiment over Twitter tennis tournament data sets and observe that our method outperforms the temporal PageRank of (Rozenshtein and Gionis 2016).
- For our new method, we give mathematical justification and perform extensive parameter analysis for properties such as convergence and adaptivity to concept drift.

## Related results

Most of the networks in nature, society, and technology change continuously. In graph theory terminology, nodes and edges get additional temporal characteristics and form a *temporal network*. We refer to (Holme and Saramäki 2012) for a recent review on various models and measures for temporal networks. The key approach is to use temporal information to create a series of snapshots and static graphs, and track dynamics for various parameters in these static graphs (Kumar et al. 2010; Rosvall and Bergstrom 2010; Sun et al. 2007). For example, one can collect all retweets on Twitter with corresponding hashtags every day to track popularity of a political party during the election period and then analyze daily changes in retweet patterns to estimate online and offline popularity of this party (Aragón et al. 2013; Gayo-Avello 2013).

To quantify the popularity of a node, several graph centrality measures have been proposed (Boldi and Vigna 2014). The definitions of centrality vary greatly and incorporate both global and local factors of a node’s location within the network. The high variability of centrality scores reflects the nature of popularity observed in real-world (Mitzenmacher 2004) and online social networks (Backstrom et al. 2012). Several models have been suggested to explain the emergence of high variability, habitually involving some variation of the preferential attachment mechanism, also extended to the dynamic setting (Hill and Braha 2010).

For temporal networks, a few generalizations of static centrality measures to dynamic settings have been suggested recently (Tang et al. 2010; Taylor et al. 2017; Kim and Anderson 2012; Grindrod and Higham 2014; Alsayed and Higham 2015). In these works, tracking centrality of a single node and determining its variability play a major role (Taylor et al. 2017), as it has been observed in the literature that centrality of nodes can change drastically from one time period to another (Braha and Bar-Yam 2006).

The above results (Taylor et al. 2017; Kim and Anderson 2012; Grindrod and Higham 2014; Alsayed and Higham 2015; Tang et al. 2010), however, cannot be used for computing and updating centrality online. The following results devise methods that are variants of our snapshot baselines: In (Taylor et al. 2017), the spectrum of a set of discrete graph snapshots is analyzed in time; however, the spectrum cannot be dynamically updated with fine time granularity, as required by our application. Similarly, in (Grindrod and Higham 2014), sequences of snapshots are considered. Finally, in (Tang et al. 2010; Kim and Anderson 2012; Alsayed and Higham 2015), degree, closeness, and betweenness are considered in dynamic graphs, bu these measures, with the exception of the degree, cannot be efficiently updated online. Note that online degree, also with time decay, is compared as a baseline method in our experiments.

In this paper we address a practically important variant of dynamic centrality: Our goal is to compute online updateable measures that can be computed from a data stream of time-stamped edges. To the best of our knowledge, the only previous such algorithms are temporal PageRank (Rozenshtein and Gionis 2016) and degree (Kim and Anderson 2012)—other measures are inefficient to update online. In our experiments, our algorithm performs well for assessing centrality in a dynamic graph, which we explain in Section Centrality in static and dynamic graphs by showing that we can incorporate temporal information while keeping dynamic update computational costs very low. In fact, temporal PageRank is based on PageRank (Page et al. 1999), while our method is based on the Katz index (Katz 1953), both of which are shown to have very similar theoretical and practical properties by (Boldi and Vigna 2014).

To our knowledge, temporal PageRank (Rozenshtein and Gionis 2016) is the only published work about temporal generalizations of PageRank. Other results focus on coarse, static snapshots such as Bonacich’s centrality (Lerman et al. 2010), or use temporal information to calculate edges of a static graph (Hu et al. 2015; Manaskasemsak et al. 2013). Finally, another line of research considers updating PageRank in dynamic or online scenarios (Bahmani et al. 2010; Bahmani et al. 2012; Kim and Choi 2015; Ohsaka et al. 2015; Sarma et al. 2011); however, in these results PageRank is considered a stationary distribution over the current, static graph. In our experiments, we will show that our temporal Katz centrality outperforms snapshot-based static measures for assessing node importance in a temporally changing environment.

## Centrality in static and dynamic graphs

Three axioms of centrality are defined in (Boldi and Vigna 2014). There is a single measure, harmonic centrality, that satisfies all three of them. Since the computation of harmonic centrality for a given node *u* involves all the distances from the node *u* in question, the measure is computationally challenging even in a static graph.

The starting point of our temporal Katz centrality measure is PageRank (Page et al. 1999), which along with the Katz index satisfies the last two axioms defined in (Boldi and Vigna 2014). PageRank is considered a success story in link analysis and listed as one of the ten most influential data mining algorithms (Wu et al. 2008). The importance of PageRank in our work has multiple reasons. On the one hand, it is widely used and has favorable properties by the axioms of (Boldi and Vigna 2014). On the other hand, temporal PageRank (Rozenshtein and Gionis 2016) is a modification of PageRank, which to the best of our knowledge is the only temporal ranking metric proposed in the literature prior to our work.

PageRank, Katz index, and temporal PageRank are all based on counting paths in the underlying networks. Next, we review the general properties of the path counting centrality metrics and temporal PageRank (Rozenshtein and Gionis 2016). Then in Section Temporal Katz centrality: our method, we describe our temporal Katz centrality measure.

### Path counting centrality metrics

As perhaps the first centrality metric based on path counting, Katz introduced his index (Katz 1953) as the summation of all paths coming into a node, but with an exponentially decaying weight based on the length of the path:

1 $$ \vec{\text{Katz}} = {\mathbf{1}} \cdot \sum\limits_{k=0}^{\infty} \beta^{k} A^{k},  $$

where $\vec {\text {Katz}}$ is the Katz index vector, *A* is the directed adjacency matrix, and *β*<1 is a constant. Hence the Katz index of a node is the weighted sum of the number of paths of different lengths *k* terminating in *u*, where the weight is *β*^*k*^:

2 $$ \vec{\text{Katz}} (u) := \sum\limits_{v} \sum\limits_{k=0}^{\infty} \beta^{k} |\{\text{paths of length} {k} \text{from} {v} \text{to} {u}\}|,  $$

The Katz index is finite only if *β*<1/|*λ*_1_|, where *λ*_1_ is the eigenvalue of *A* with largest absolute value (Katz 1953). Since 1/|*λ*_1_| is often very small, around 0.05 in our graphs, the relative weight of a length two path stays very small compared to a single edge. In order to be able to use larger values of *β*, we introduce the truncated Katz index as

3 $$ \vec{\text{Katz}}^{[K]} = {\mathbf{1}} \cdot \sum\limits_{k=0}^{K} \beta^{k} A^{k}.  $$

Note that $\vec {\text {Katz}}^{[\infty ]} = \vec {\text {Katz}}$.

By the basic definition, PageRank is normally considered to be the static distribution of a random walk with damping (Page et al. 1999). In order to compare PageRank and the Katz index, and to motivate online update rules, we use the result of (Fogaras et al. 2005), who show—and use as an efficient algorithm—that PageRank is equal to the path counting formula

4 $$ \vec{\text{PageRank}} = {\mathbf{1}} \cdot \frac{c}{N} \cdot \sum\limits_{k=0}^{\infty} (1-c)^{k} M^{k},  $$

where *c* is the damping constant and *M* is the random walk transition matrix. In other words, *M* is the outdegree normalized adjacency matrix: *M*=(*K*^−1^*A*)^*T*^ where *K* is a diagonal matrix with the outdegrees in the diagonal.

### Temporal PageRank

In (Rozenshtein and Gionis 2016), temporal PageRank, a dynamic variant of PageRank, is defined as follows. In a dynamic graph, edges are time-stamped and can appear multiple times. The main idea is to aggregate **time respecting temporal walks**

5 $$ z = \left(u_{0},u_{1},t_{1}\right),\left(u_{1},u_{2},t_{2}\right),\cdots,\left(u_{j-1},u_{j},t_{j}\right); \hspace{0.7cm} t_{i-1}\leq t_{i}.  $$

ending in a certain node, as illustrated in Fig. 1, to compute its temporal centrality. In such a walk, they model an information flow from the start node *u*_0_ to the destination *u*_*j*_ by passing along edges that arrive subsequently in time.

For each edge (*u*_*i*−1_,*u*_*i*_,*t*_*i*_) in walk *z*, they assign the transition weight as *β*^*k*^, where *β*<1 is a decay constant and *k* is the number of edges (*u*_*i*−1_,*y,t*^′^) that appear after the previous edge but not later than the present edge in the walk, that is, *t*_*i*−1_<*t*^′^<*t*_*i*_. They incorporate this weight assignment in formula (4); for full details, see (Rozenshtein and Gionis 2016).

Intuitively, their notion of edge transition weight decays exponentially with the number of possible continuations of the temporal walk at node *u*_*i*−1_. The more edges appear before (*u*_*i*−1_,*u*_*i*_,*t*_*i*_), in their model it is exponentially less likely that the information is sent along the given edge—and not another edge that appears earlier.

The main problem with the above path counting algorithm is that it overvalues nodes with low activity. Consider a node that communicates to ten contacts in a few minutes. The tenth contact will only receive a propagated score proportional to *β*^−10^. By contrast, if another node sends only one message per day, the neighbor receives the full score even though the information may already be highly outdated.

One key motivation of the above definition for temporal PageRank is that it possesses a computationally low cost update algorithm. While it is tempting to modify the weight formula to incorporate the actual time elapsed, the stream-based computation of such a modified temporal PageRank becomes unclear.

## Temporal Katz centrality: our method

We define our temporal Katz centrality measure over the stream of edges arriving in time from a dynamic network. Our goal is to specify a metric that is based on the weighted sum of time respecting walks, updateable by the edge stream, and that can incorporate the actual elapsed time in the weights of the walks.

To motivate our new method, we reconsider the temporal PageRank (Rozenshtein and Gionis 2016) edge transition weight rule: Weight *β*^*k*^ is assigned to an edge *uv* in a path where *k* is the number of edges that appear after the previous edge entering *u* but not later than the appearance of edge *uv*. The definition involves time decay in an indirect way through a combination with the activity of the nodes. As an advantage, the definition guarantees that the weight will incur the degree normalization required in the PageRank Eq. (4), and hence temporal PageRank will converge to static PageRank if edges are played several times in random order. As a disadvantage, the notion of time is difficult to directly capture in the temporal PageRank algorithm. The more time elapses before the next edge appears, the more other edges have the chance to appear in between. However, this notion also depends on the activity of the node in question, and longer delays are penalized less at inactive nodes compared to active nodes.

**Fig. 2:**
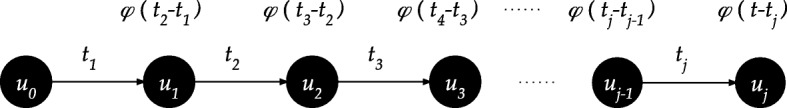
Edge weights along a temporal walk at time *t*

We define **temporal Katz centrality** by introducing a natural, purely time-dependent edge transition weight *φ*(*τ*), which is an arbitrary function of the time elapsed since the previous edge in a path. Intuitively, we define a time dependent decay for each edge, as shown in Fig. 2. We will use the edge decay values to compute an aggregated freshness of the information flow along a given path, which we will in turn aggregate for the final nodes of the paths.

1. Temporal Katz centrality is the weighted sum of all time respecting walks that end in node *u*,
  6 $$ r_{u} (t) := \sum\limits_{v} \sum\limits_{\substack{\text{temporal paths } {z} \\ \text{from } {v} \text{to} {u}}} \Phi(z,t)  $$
  where *Φ*(*z,t*) is the weight of walk *z* at time *t*. Truncated temporal Katz centrality is defined similar to Eq. (3) by restricting to walks of length at most *K*.
2. For a temporal walk as in Eq. (5) where edges appeared at (*t*_1_,*t*_2_,…,*t*_*j*_), we define weight *Φ*(*z,t*) as
  7 $$ \Phi(z,t):= \prod_{i=1}^{j} \varphi(t_{i+1}-t_{i}),  $$
  where *φ* is a time-aware weighting function, and for *i*=*j* we let *t*_*j*+1_:=*t*.
3. Hence *Φ*(*z,t*) is the product of individual edge transition weights *φ*(*t*_*i*+1_−*t*_*i*_) as seen in Fig. 2. The last term of the product *φ*(*t*−*t*_*j*_) captures the delay between present time *t* and the appearance of the last edge in the path.

By combining Eqs. (6)–(7) temporal Katz centrality can be considered a variant of the Katz index Eq. (2), in which time respecting paths are weighted by *Φ*(*z,t*):

8 $$ r_{u} (t) := \sum\limits_{v} \sum\limits_{\substack{\text{temporal paths } {z} \\ \text{from } {v} \text{to} {u}}} \prod_{i=1}^{j} \varphi(t_{i+1}-t_{i}).  $$

By using different edge weight functions, we cover two important special cases for temporal Katz centrality:

- If *φ*(*τ*):=*β* is constant, we obtain a variant of the Katz Eq. (2) with summation for temporal paths instead of all paths irrespective of time.
- In another special case, *φ*(*τ*):=*β*· exp(−*cτ*). Since *φ* is an exponential function, *φ*(*a*)·*φ*(*b*)=*φ*(*a*+*b*). Hence the path weight in (7) becomes
  9 $$ \Phi(z,t)= \beta \exp\left(-c\left[t- t_{j}\right]\right) \ldots \beta \exp\left(-c\left[t_{2} - t_{1}\right]\right) = \beta^{|z|} \exp \left(-c\left[t- t_{1}\right]\right),   $$
  that is, it involves a Katz-style decay proportional to the length of the path, combined with an exponential decay depending on the time elapsed since the first interaction *t*_1_ over the path occurred. This weight is capable of capturing the temporal decay of information spreading and propagation.

### Update formula

**Fig. 3:**
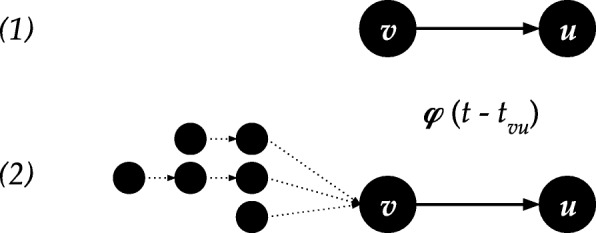
At time *t* when edge *vu* becomes active, (1) a new walk appears starting from *v*, and (2) each time respecting walk that ended in *v* continues to *u*

In this section, we show how we can maintain temporal Katz centrality *r*_*u*_ for each node *u*, which is the sum of temporal paths *z* as in Eq. (5) with weight *Φ*(*z,t*) as in (7). We base our analysis below on the fact that the sum of all temporal paths to *u* can be derived by using the number of temporal paths ending at the in-edges of *u*. As seen in Fig. 3, if edge *vu* appears at time *t*_*vu*_, the future centrality of node *u* at time *t* increases as

1. a new time respecting walk appears that starts from *v* and has weight *φ*(*t*−*t*_*vu*_),
2. for each time respecting walk that ended in *v* at *t*_*vu*_, a new walk with the new edge *vu* appears. The total weight of paths that ended in *v* is *r*_*v*_(*t*_*vu*_), hence the weight of the new walks is *r*_*v*_(*t*_*vu*_)·*φ*(*t*−*t*_*vu*_).

Adding up the weight of the two types of new walks, we get

10 $$ r_{u}(t) = \sum\limits_{vu \in E(t)} \left (1 + r_{v}(t_{vu}) \right) \varphi(t - t_{vu}),  $$

where *E*(*t*) is the multi-set of edges appearing no later than *t*. Based on the above recursive formula, if edge *vu* appears at time *t*_*vu*_, it increases the future centrality of node *u* by (1+*r*_*v*_(*t*_*vu*_))*φ*(*t*−*t*_*vu*_). The increase of the centrality of *u* can be computed by maintaining the values *t*_*vu*_ and *w*_*vu*_:=1+*r*_*v*_(*t*_*vu*_). The algorithm for updating temporal Katz centrality is hence the following:

- For each node *u*, we initialize temporal Katz centrality *r*_*u*_ as constant 0. For each edge *vu*, we maintain the edge weight *w*_*vu*_ and the time of appearance *t*_*vu*_, initially all set to 0 and −*∞*, respectively. We let *E*(*t*) denote the multi-set of edges that appeared before time *t*.
- Next, we consume the stream of edges *vu* and we update *r* and *w* as follows. First we calculate the current value of *r*_*v*_ as
  11 $$ r_{v}:= \sum\limits_{zv \in E (t)}{w_{zv} \cdot \varphi(t-t_{zv})}.  $$
  Here *E*(*t*) is a multi-set, and each past occurrence of edge *zv* is counted separately, with different *t*_*zv*_ and hence different decay. Note that when edge *vu* appears, *t*=*t*_*vu*_.
- Then we add a new edge *vu* to the multi-set of edges with *w*_*vu*_:=*r*_*v*_+1 to propagate the centrality score along edge *vu*, and set *t*_*vu*_:=*t*.
- The above algorithm can also be applied to update truncated temporal Katz centrality by the following modification: We maintain an array $w_{vu}^{[k]}$ for *k*=1,…,*K* for each edge in the multi-set *E*(*t*), and set
  12 $$\begin{array}{@{}rcl@{}} w_{vu}^{[1]}&:=& 1 \\ w_{vu}^{[k]}&:=& 1 + \sum\limits_{zv \in E (t)}{w_{zv}^{[k-1]} \cdot \varphi(t-t_{zv})} \quad\text{ for } 1<k\le K. \end{array} $$
  13 $$\begin{array}{@{}rcl@{}} r_{u}^{[k]} &:=& \sum\limits_{vu \in E (t)} w_{vu}^{[k]} \cdot \varphi(t-t_{vu}) \end{array} $$

Time ordering is consistent with information propagation: For a path of three nodes *u*, *v*, and *z*, we can propagate a certain share of the *r*_*u*_ score along edge *vz* only by first propagating along *uv*; hence *uv* must appear before *vz*.

To relate temporal Katz centrality to (online) PageRank, notice the difference of the Katz and PageRank path counting formulas (1) and (4). In Katz, the exponential decay is applied to powers of the binary valued adjacency matrix *A*, while in PageRank, to the degree normalized random walk matrix *M*.

Observe the lazy behavior of the algorithm: Ranks are updated only for the tail *v* of each new edge *vu*. We assign based on the centrality of *v* *r*_*v*_+1, as the weight *w*_*vu*_. If we query the rank of *u*, we propagate *r*_*v*_ along edges *vu*; however, we add a time decay to account for the freshness of the edges *vu*: More recent edges propagate scores with higher intensity.

### Time complexity

The time complexity of maintaining *r*_*u*_ by formula (11) is linear in the degree of *u*. We can further improve the online update complexity to constant time per update if *φ* satisfies *φ*(*a*+*b*)=*φ*(*a*)·*φ*(*b*). In this case, it is easy to see that at query time *t*, we can recompute *r*_*u*_ by the actual time *t* in formula (11) as

14 $$ r_{u}:= r_{u} \cdot \varphi(t-t_{u}),  $$

where *t*_*u*_ is the last time node *u* was updated.

We can combine formulas (11), (10) and (14) to update *r*_*u*_ for each new edge (*vu*) by

15 $$\begin{array}{@{}rcl@{}}  r_{v} &:=& r_{v} \cdot \varphi(t-t_{v});\\  r_{u} &:=& r_{u} \cdot \varphi(t-t_{u}) + (r_{v} +1) \cdot \beta;\\ t_{u} &:=& t, \hspace{0.22 cm} t_{v} := t, \end{array} $$

Querying the centrality score of a single node can be served in constant time by formula (14). Hence computing a centrality top list can be done in time linear in the number of vertices. For the special case when *φ*(*t*)=1, the scores change only when formula 15 is applied, hence the scores can be stored, for example, in a heap to quickly access the maximum score. In other cases, we can deploy heuristics such as (Teflioudi et al. 2015) to quickly find *u* that maximizes the product (14); however, such an optimization is out of scope in this paper.

Overall, for the decay functions *φ* used in our experiments, the time complexity of our method is identical to that of time decayed degree. In the special case of *φ*=1, our time complexity is equal to that of static degree, while for other decay functions, we can bring the running time very close to static degree by applying heuristics to find the maximum of a product (Teflioudi et al. 2015).

**Fig. 4:**
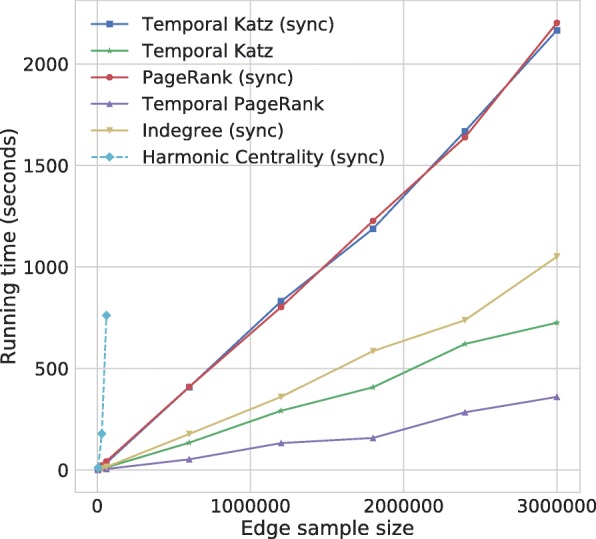
The running time of temporal PageRank, static PageRank, static indegree, harmonic centrality, and temporal Katz centrality with and witout synchronizing with time decay as in Eq. (14), measured over random Barabási–Albert graphs with sizes as in Table 1. All static centrality measures are considered to be synchronized

We experimentally compared the running time of our method with static indegree, static PageRank, temporal PageRank, and harmonic centrality in Fig. 4. We generated random Barabási–Albert graphs (Barabási 2009) by the barabasi_albert_graph method of the networkx Python package^1^ and constructed temporal graphs by using a 10% sample of the edges in random order. We split the temporal graph into ten equal sized slices and computed all node centrality values at the end of each of the ten slices. The size of the graphs are found in Table 1.

**Table 1:** The size of the random Barabási–Albert graphs generated for the scalability experiments

Nodes	Edges	Edge sample size
10 000	59 982	5 998
50 000	299 982	29 998
100 000	599 982	59 998
1 000 000	5 999 982	599 998
2 000 000	11 999 982	1 199 998
3 000 000	17 999 982	1 799 998
4 000 000	23 999 982	2 399 998
5 000 000	29 999 982	2 999 998

As seen in Fig. 4, except for harmonic centrality, all algorithms scale linear with the number of edges. For our temporal Katz centrality algorithm, more than half of the running time is consumed by multiplying the centrality values by the time decay as in Eq. (14) at the time of reading the observations. Hence we also report the running times of our method without time decay synchronization at the end of the time frames. Overall, we observed that the running time of these methods show implementational rather than algorithmic differences.

### Normalization for numeric stability

Next we describe how to normalize the temporal Katz centrality scores throughout the computations for numeric stability. The main reason is that in our experiments, the values often resulted in numeric overflow for the best performing values of *β*. Since for a ranking method, the actual values of the score are indifferent, and only the rank order matters, we can apply any method to normalize temporal Katz centrality. The main challenge is that the normalization method must also be online updateable.

First, we discuss the numerical importance of normalizing temporal Katz centrality. Katz index (1) converges only if *β* is less than the inverse of the largest eigenvalue of *A* (Katz 1953). Typical maximal values of *β* for real graphs are in the range of 0.01–0.05, which gives small weight for longer paths. By contrast, temporal Katz centrality performed best in our experiments for detecting important nodes of the network for much larger values *β*. For the high values of *β*, the centrality scores quickly grow to infinity, as it happened in our experiments. For this reason, next we propose a method for normalizing temporal Katz centrality.

To normalize the centrality scores, it is sufficient to maintain the sum of the raw scores. Given the sum, we can always divide raw scores by the sum to obtain the normalized values. In order to ensure that the raw values and the sum do not grow unbounded, we have to periodically apply the normalization to all values. Unfortunately, synchronized normalization of all values is not possible in the data streaming model. Instead, we apply lazy normalization and maintain the time-stamped history of the multipliers. Whenever we touch a centrality value, we first check its time stamp to see if pending normalization steps need to be taken first before using the value.

Finally, we describe the algorithm to maintain the sum of the centrality scores. Instead of the lazy algorithm in Section Update formula, which updates centrality *r*_*u*_ only when a new edge *uv* appears that will later propagate the value of *r*_*u*_ to node *v*, we theoretically maintain the actual score at every time instance. First, for every clock tick of time *τ*, we multiply each *r*_*u*_, and hence also the sum, by *e*^−*τ*^ as in Eq. (14). Second, we consider an event when edge *uv* appears. At this time, the value of *r*_*u*_ is computed by the update Eq. (11). This new edge propagates the score *r*_*u*_ to *v* and thus increases *r*_*v*_ by *r*_*u*_. Hence for all new edges, the increase of the sum at the time edge *uv* appears is *r*_*u*_ measured at that time. To maintain the total sum of the centrality scores, all is required is to add up *r*_*u*_ in Eq. (14) whenever it is applied by the update algorithm, and multiply by *e*^−*Δt*^ at every clock tick of time *Δt*.

### Convergence properties

Let us assume that we sample a sequence of *T* edges from a graph with edge set of size *E*. We intend to compute the expected value of temporal Katz centrality over the sampled edge stream, under the assumption that the activation of the links of the underlying graph is random. We give estimates on the number of times a given path is expected to appear in time respective order, which yields in convergence theorems for temporal Katz centrality to an expression similar to the Katz index. Note that we assume that sampling is done in a uniform way over time, hence in what follows, time *t* corresponds to the number of sampled edges in the process.

#### **Theorem 1**

Let us compute (truncated or normal) temporal Katz centrality with *Φ*(*z,t*)=*β*^|*z*|^ (no decay). If we sample a sequence of *T* edges from an edge set of size *E*, the expected value of temporal Katz centrality is

16 $$ \vec{{\text{TemporalKatz}}} = {\mathbf{1}} \cdot \sum\limits_{k=0}^{K} \beta^{k} A^{k} {T \choose k} \cdot E^{-k} \simeq {\mathbf{1}} \cdot \sum\limits_{k=0}^{K} \beta^{k} A^{k} (T/E)^{k} / k!.  $$

#### *Proof*

The expected number of times the edges of a given path of length *k* appear in a given order, in an edge sample of size *T* can be computed as

17 $$ s_{T, k} = {T \choose k} \cdot E^{-k},  $$

since a given edge has a probability of 1/*E* to appear at a given position in the sequence of *T* edges. To complete the proof, observe that by Eq. (8), temporal Katz centrality is

18 $$ \vec{{\text{TemporalKatz}}} = {\mathbf{1}} \cdot \sum\limits_{k=0}^{\infty} \beta^{k} A^{k} \cdot s_{T, k} = {\mathbf{1}} \cdot \sum\limits_{k=0}^{K} \beta^{k} A^{k} {T \choose k} \cdot E^{-k}  $$

□

#### **Theorem 2**

Let us sample a sequence of *T* edges from an edge set of size *E*. Let us compute (truncated or normal) temporal Katz centrality with exponential weighting, *φ*(*τ*):=*β* exp(−*cτ*). Then as *T*↦*∞*, the limit of the expected value of temporal Katz centrality is

19 $$ \vec{{\text{TemporalKatz}}} = {\mathbf{1}} \cdot \sum\limits_{k=0}^{K} A^{k} \left(\frac{\beta}{E} \right)^{k} \left (\frac{1}{e^{c}-1} \right)^ k.  $$

In particular, if *c*=*c*^′^/*E* with *c*^′^≪*E*, then the expected value of temporal Katz centrality is approximately

20 $$ \vec{{\text{TemporalKatz}}} = {\mathbf{1}} \cdot \sum\limits_{k=0}^{K} A^{k} \left(\frac{\beta}{c^{\prime}} \right)^{k}.  $$

#### *Proof*

We intend to compute

21 $$ \vec{{\text{TemporalKatz}}} = {\lim}_{T \rightarrow \infty} {\mathbf{1}} \cdot \sum\limits_{k=0}^{K} A^{k} s_{T,k} = {\mathbf{1}} \cdot \sum\limits_{k=0}^{K} A^{k} {\lim}_{T \rightarrow \infty} s_{T,k} {,}  $$

where *s*_*T,k*_ denotes the expected total weight of a given path of length *k* in an edge sample of size *T*.

**Fig. 5:**
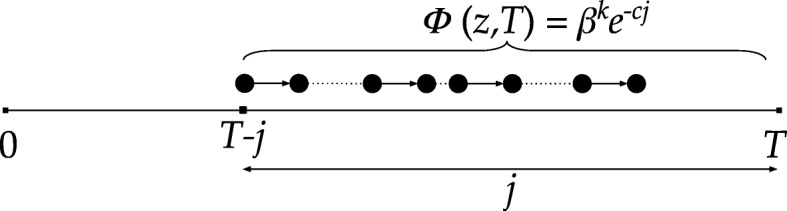
Explanation of Theorem 2. Each occurrence of a given path of length *k* that starts at time *T*−*j* has the same weight *β*^*k*^ exp(−*cj*)

Let us consider a given path of length *k* starting at time *t*_1_=*T*−*j* as seen in Fig. 5. Each possible occurrence of the path starting at the same time *t*_1_=*T*−*j* has the same weight *Φ*(*z,T*)=*β*^*k*^*e*^−*cj*^ (see (7) and (9)). Since we fix the first edge of these occurrences, by Eq. (17), the expected number of the occurrences is $\frac {1}{E^{k}}{j-1 \choose k-1}$. As a result, the expected total weight of a given path of length *k* is

22 $$ s_{T,k} = \beta^{k} \frac{1}{E^{k}} \sum\limits_{j=k}^{T}{j-1 \choose k-1} e^{-cj}.  $$

Since $\sum \limits _{n=m}^{\infty } {n \choose m} x^ n = x^{m} / (1-x)^{m+1}$,

23 $$\begin{array}{@{}rcl@{}}  {\lim}_{T \rightarrow \infty} s_{T,k} &=& {\lim}_{T \rightarrow \infty} \left (\frac{\beta}{E} \right)^{k} \sum\limits_{j=k}^{T}{j-1 \choose k-1} e^{-cj}\\ = \left (\frac{\beta}{E} \right)^{k} e^{-c}\sum\limits_{j=k}^{\infty}{j-1 \choose k-1} e^{-c(j-1)} \end{array} $$

24 $$\begin{array}{@{}rcl@{}} = \left (\frac{\beta}{E} \right)^{k} \frac{e^{-ck}}{(1 - e^{-c})^{k}} = \left (\frac{\beta}{E} \right)^{k} \frac{1}{(e^{c}-1)^{k}}.  \end{array} $$

Hence

25 $$ \vec{{\text{TemporalKatz}}} = {\mathbf{1}} \cdot \sum\limits_{k=0}^{K} A^{k} {\lim}_{T \rightarrow \infty} s_{T,k}= {\mathbf{1}} \cdot \sum\limits_{k=0}^{K} A^{k} \left (\frac{\beta}{E} \right)^{k} \left (\frac{1}{e^{c}-1} \right)^ k.  $$

If *c*=*c*^′^/*E* with *c*^′^≪*E*, then *c*^′^/*E*<<1 and $e^{c^{\prime }/E} \approx 1 + {c^{\prime }/E}$; hence

26 $$ \vec{{\text{TemporalKatz}}} = {\mathbf{1}} \cdot \sum\limits_{k=0}^{K} A^{k} \left (\frac{\beta}{E} \right)^{k} \left (\frac{1}{1 + c^{\prime}/E -1} \right)^ k = {\mathbf{1}} \cdot \sum\limits_{k=0}^{K} A^{k} \left (\frac{\beta}{c^{\prime}} \right)^{k}.  $$

□

There is always a certain amount of fluctuation in temporal centrality as the effect of the most recently selected edges. We can compute the expected increase for the weight of paths that end with the most recently selected edge.

For the case with no decay, the additional count is the number of times the length *k*−1 prefix appears, which is *s*_*T*−1,*k*−1_. The increase is approximately a multiplicative (1+*k*/*E*) factor, which may be large for a large *k*; however, the weight of long paths is diminishing exponentially as *β*^*k*^.

For the case with decay, the increase is given by Eq. (24) applied with *k*−1 instead of *k*, which approximately gives an expected multiplicative increase (1+1/(*Ee*^−*c*^)), which is approximately 1+*c*^′^ for the special case of Theorem 2.

## Twitter Tennis data sets

We compiled two separate tweet collections, *RG17* for Roland-Garros 2017, the French Open Tennis Tournament, and *UO17* for US Open 2017, the United States Open Tennis Championship. The events took place between May 22 and June 11 as well as August 22 and September 10, respectively. We assessed the temporal relevance of centrality measures by using the list of players of different days as ground truth. We gathered data with the Twitter Search API, by using the following two separate sets of keywords:


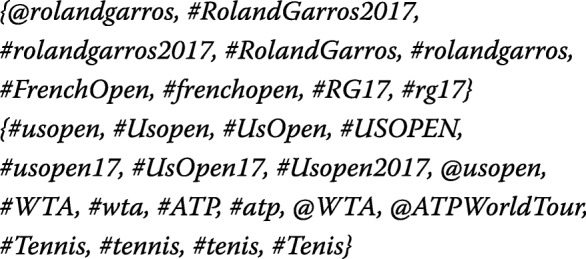


**Fig. 6:**
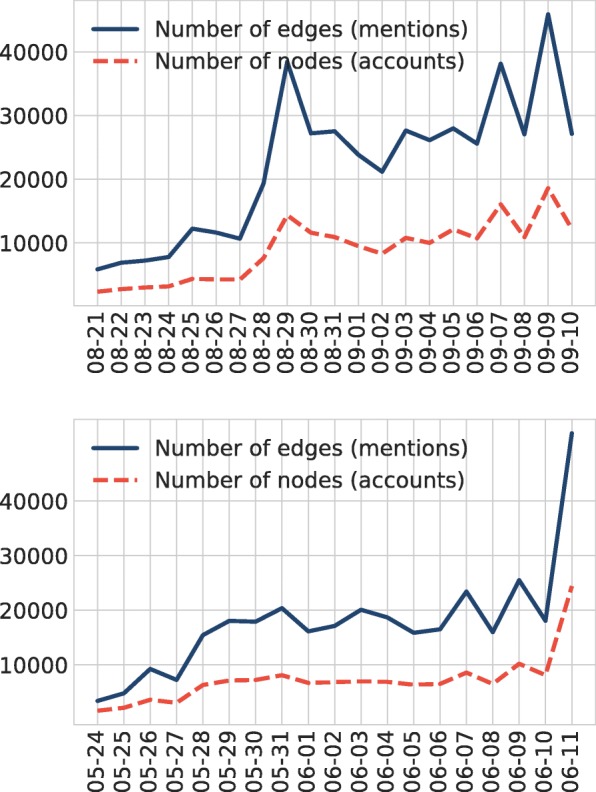
Number of nodes and edges in the UO17 (top) and RG17 (bottom) mention graphs. During the qualifiers the number of interactions is low. Then user activity increases as the championship starts from Sept 28 or May 28 respectively. For UO17 the two bursts on September 7 and 9 are related to Women’s Singles semi-final and final. A similar behavior can be observed for RG17 due to Men’s Singles finals on June 7–9–11

The RG17 data covers the events of the championship starting May 24 with 444,328 tweets, 815,086 retweets, and 336,234 time-stamped mentions. The UO17 data consists of 636,810 tweets, 1,048,786 retweets, and 482,061 mentions. The daily distribution of mentions is shown for both tennis events in Fig. 6. Note that we imposed no language restrictions on the text of the tweets during the data collection process.

We measure the performance of centrality measures by means of comparison with the official schedule of the tournaments. The daily timetables are accessible in HTML file format and contain the following information for each tennis game:

- Full names of the participating players (two for singles and four for doubles games)
- Approximate time of the game during the day (e.g.: after 11:00, not before 15:00, etc.)
- Category and round identifier of the game (e.g. Women’s Singles—Round 1, Men’s Singles—Final)
- Court name, where the game took place (e.g. Grandstand, Arthur Ashe Stadium, etc.)
- Information about whether the game was canceled, resumed from a previous day, or the final result if completed.

Based on the approximate time of the games, we consider a player *active* for a given day if he or she participated in a *completed game*, a *canceled game*, or a *resumed game* on the same day. All of these events are expected to cause a social media burst.

One of the most time-consuming parts of our measurement was to assign Twitter accounts to tennis players. The total number of professional participants is 798 for US Open and 698 for Roland-Garros. Unfortunately, many of the players have no Twitter accounts.

We assigned players to accounts by the Twitter Search API’s people endpoint; however, the API was sometimes unable to identify the accounts of the active players.

In case the people API endpoint failed to return the account of a player, we considered the *account name* (e.g. @rogerfederer, @RafaelNadal) and *name* (e.g. “RafaNadal” for the account @RafaelNadal). Using edit distance, for each player we automatically selected accounts where the *account name* or the displayed *name* is very similar to the full name. Note that the same player often has multiple Twitter accounts, especially the popular players, who usually have official sites and distinct accounts for fans with different nationalities. As a last step, we excluded fake assignments such as @AndyMurray and @DominicThiem by manual verification.

In order to match accounts and player names, we first listed the accounts that have minimum edit distance from a given player’s name. We removed whitespaces and transformed all characters to lower case. Since name matching can lead to false player-account pairs, we manually searched the lists of different edit distance values to find valid player account matches. We first considered screen names, and in case there was no match, we continued with account names.

**Fig. 7:**
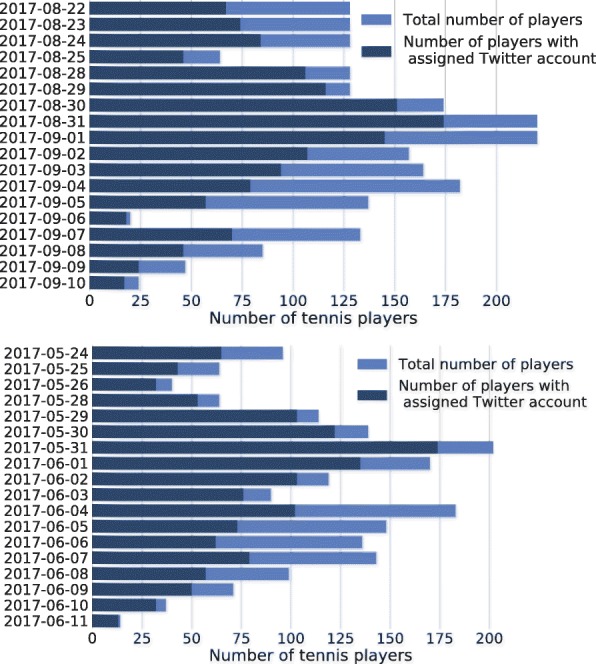
The number of players active on a given day and the number of them with identified Twitter accounts. Top: UO17; bottom: RG17. Days with no tennis game between the qualifiers and the championship (Aug 26-27 and May 27, respectively) are not shown

Using the above semi-automatic procedure, we managed to find Twitter accounts for 58.4% of the US Open players, as seen in Fig. 7. We achieved better player coverage of 64.2% for Roland-Garros.

**Table 2:** Summary of the data sets used

	Edges	Nodes	Days
Students	10,000	1654	121
Facebook	10,000	4752	104
Enron	6251	1944	892
Tumblr	7645	1757	89
UO17	482,061	106,920	21
RG17	336,234	78,095	19

## Unsupervised evaluation

In addition to the data with ground truth of the previous section, we used the data sets of (Rozenshtein and Gionis 2016) for unsupervised analysis (see Table 2). These small temporal networks (Students, Facebook, Enron, Tumblr) have no more than 10,000 edges^2^, as seen in Table 2.

### Stability vs. changeability

**Fig. 8:**
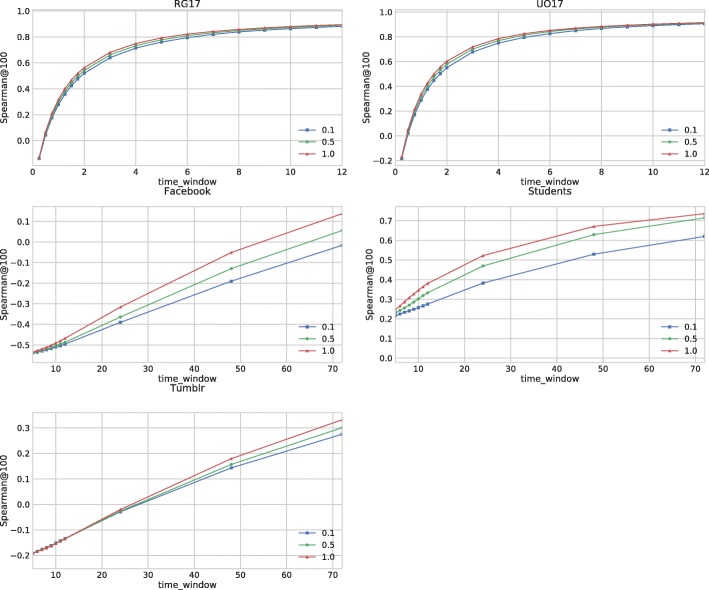
Average Spearman correlation between temporal Katz centrality scores of adjacent snapshots. Daily snapshots are used for Facebook, Students and Tumblr data sets, and hourly snapshots are used for RG17 and UO17 Twitter collections. The correlation is presented for *β* values 0.1,0.5,1.0 and several time decay intensity

We assess the amount of variability of temporal Katz centrality in time, depending on the parameters *β* and the time decay exponent to exhibit the speed of focus shift in daily interactions. We use the weight function *φ*(*τ*)=*β*·2^−*cτ*^; *c* can be considered as the half-life of the information sent over an edge. We update temporal Katz centrality after each edge arrival, and compute the top 100 nodes with highest centrality scores for each snapshot. We generate the lists at the beginning of each day for the small data sets of (Rozenshtein and Gionis 2016), and each hour for our Twitter collections RG17 and UO17. Spearman correlation is calculated between lists of adjacent snapshots, for different values of *c* and *β*, as shown in Fig. 8.

Our measurements show that the similarity between adjacent lists depends on two different factors. We can turn temporal Katz centrality more static by using longer half-life in the decay. If the half-life is short, we even get negative correlations as the number of nodes present in both lists decreases. Another option is to use larger *β*. By increasing *β*, the contribution of long walks will be more relevant, which cannot be dominated by recently added edges as easily as for a small *β*. The two approaches can also be used in combination. We observed the highest similarity using *β*=1.0 with large half-life value.

### Adaptation to concept drift

Rozenshtein et al. (2016) showed that temporal PageRank can adapt to the changes in the edge sampling distribution over semi-temporal networks. We conducted similar measurement for temporal Katz centrality on the same data sets: We created concept drift by changing the sampling distribution that generates the temporal graphs and measuring how quickly the different methods get closer to the static centrality measure of the new distribution.

We created concept drift by changing the sampling distribution that generates the edge stream. We measured how quickly different temporal centrality measures converge to the static centrality measure of the new distribution.

In our experiment for concept drift adaptation, we randomly selected 500 nodes as a base graph and formed three overlapping subsamples of 400 nodes each. Similar to the approach in (Rozenshtein and Gionis 2016), we formed a temporal edge stream of three segments corresponding to the three subsamples, in each segment selecting 10,000 random edges from the corresponding subsample. We compute temporal PageRank and temporal Katz centrality by assuming that a new edge in the stream appears in each time unit. In other words, we measure the elapsed time *τ* by the number of edges in the stream.

We computed weighted Kendall tau (Vigna 2015) rank distance between temporal Katz centrality and static Katz index restricted to the nodes of the actual subsample. This results in concept drift with three different versions of the static centrality score corresponding to the three time periods. By using weighted Kendall tau for measuring concept drift adaptation, we put more emphasis on nodes with high centrality compared to (unweighted) Kendall tau. For the same reason, we use the asymmetric version as in (Vigna 2015, Section 5.1) by using the weight of 1/rank for the static Katz index and zero for the online methods. By this choice, Kendall tau measures the distance from the Katz index acting as ground truth.

**Fig. 9:**
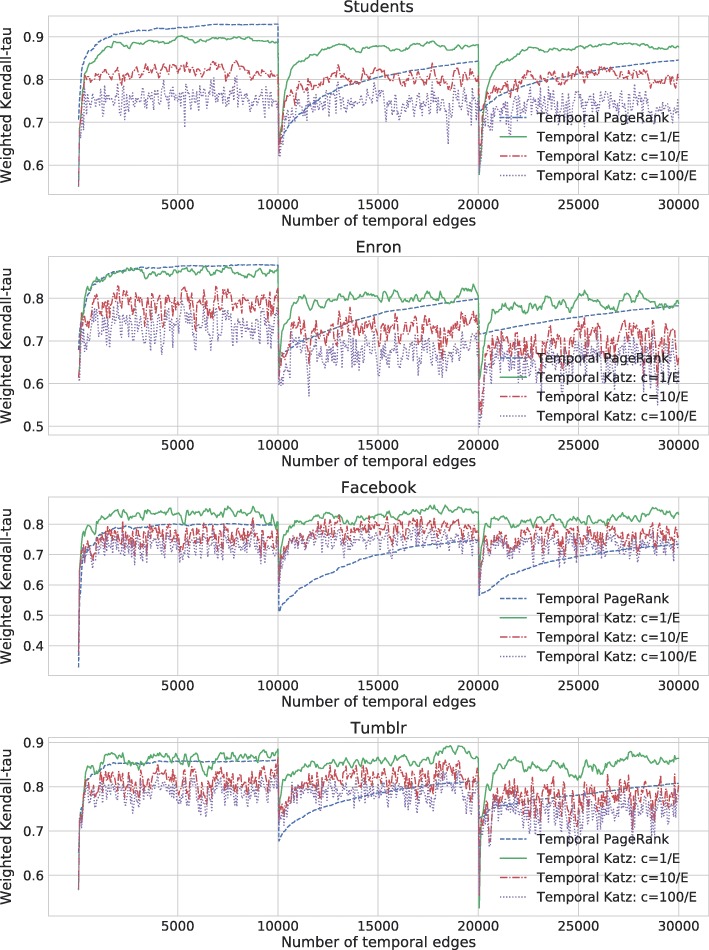
Weighted Kendall tau rank distance of static Katz index and online methods by sampling to simulate concept drift over Students, Enron, Facebook and Tumblr data. Static Katz index has *β*=0.01. The Weighted Kendall tau curves for temporal Katz centrality with $c=\frac {1}{|E|}$ are green, with $c=\frac {10}{|E|}$ are red, with $c=\frac {100}{|E|}$ are purple, and for temporal PageRank are blue dashed. Noise in temporal Katz centrality is due to the effect of the most recently selected edges. The two vertical bars mark the time of the concept drift, when a new sampling distribution is used to generate the temporal edges

In Fig. 9, we evaluated our model for various values of the exponential decay against the Katz index with *β*=0.01. The results show that in case of weak decay $c=\frac {1}{|E|}$, temporal Katz centrality becomes similar to static Katz index as the graphs evolve, which is in accordance to Theorem 2 stating that temporal Katz centrality converges to an expression similar to the static Katz index. On the contrary, strong decay shifts the focus of temporal centrality towards the recently sampled edges, thus correlation decrease for $c=\frac {10}{|E|}$ and $c=\frac {100}{|E|}$. Also note the noise in temporal Katz centrality rank distance curves due to the effect of the most recently selected edges, as described in Section Convergence properties.

To summarize our experiments in Fig. 9, we considered the behavior of temporal Katz centrality with different parameters as well as temporal PageRank after the two changes in sampling distribution marked by vertical bars in the Figure. We observed that temporal PageRank forgets the old distribution very slow, while temporal Katz centrality very quickly becomes similar to the new static distribution. The best parameter for temporal Katz centrality is a weak decay $c=\frac {1}{|E|}$, which is still sufficient to forget the old distribution but gives less fluctuation compared to the very highly adaptive, stronger decay versions with larger values of *c*.

## Supervised evaluation

In this section, we quantitatively analyze the relevance of temporal centrality measures over the UO17 and RG17 Twitter collections. We compare the relevance of temporal Katz centrality to temporal PageRank and other *online* and *static* baseline methods described in Section Baseline metrics.

To evaluate online metrics, we perform continuous update as the new edges arrive, by considering our data as a time-ordered edge stream. For the static metrics, we consider different graph snapshots. For each centrality measure, we compute the list of the nodes with the highest centrality in *each hour*. We use NDCG (Al-Maskari et al. 2007) for evaluation, defined as follows. For a list of length *k* that contains the top nodes sorted by their centrality metric, we compute the weighted sum of node relevances:

27 $$ \text{DCG}@k = \sum\limits_{i=1}^{k} \frac{\text{rel} (n_{i})}{\log_{2} (i+1)},   $$

where *n*_*i*_ is the node at position *i* in the list and *rel*(*n*_*i*_) is its relevance: An account *n*_*i*_ is relevant if it corresponds to a tennis player that participated in the tournaments of the current day:

28 $$ \text{rel}(n_{i}):=\left\{\begin{array}{ll} 1, \text{ \(n_{i}\) plays on the current day }\\ 0, \text{ otherwise.} \end{array}\right.  $$

Finally, NDCG is the normalized version of DCG:

29 $$ \text{NDCG@K} = \frac{\text{DCG@K}}{\text{IDCG@K}},  $$

where IDCG is the “ideal” DCG we get by ordering the nodes according to their true relevance.

### Baseline metrics

We compare temporal Katz centrality to *online* (or time-aware) and *static* (or batch) metrics. Online metrics are updated after the arrival of each edge. By contrast, static metrics are only updated once in each hour. At hour *t* a static metric is computed on the graph constructed from edges arriving in time window [*t*−*T,t*] from the edge stream. For each baseline, we experimentally select the best value of *T*.

We consider four *static* centrality measures as baseline:

- *PageRank* (Page et al. 1999): We set *α*=0.85, and 50 iterations.
- *indegree*: We calculate the indegree of each node in time window [*t*−*T,t*] by counting each edge once, that is, without multiplicity.
- *negative* *β*-*measure* (Boldi and Vigna 2014): The normalized version of indegree, for node *u*
  30 $$ \sum\limits_{z \in N_{in}(u)} \frac{1}{\text{outdegree}(z)},  $$
  where *N*_*in*_(*u*) denotes the in-neighbors of *u*.
- *harmonic centrality* (Boldi and Vigna 2014): For node *u*
  31 $$ \sum\limits_{z \not= u} \frac{1}{d(z,u)}.  $$

Furthermore, we compare temporal Katz centrality with two *online* metrics, temporal PageRank (Rozenshtein and Gionis 2016) and decayed indegree.

- *temporal PageRank:* We set *α*=0.85 and *β*∈{0.001,0.01,0.05,0.1,0.5,0.9} for transition weight.
- *decayed indegree:* Using the notations of Section Update formula, the decayed indegree of node *u* at time *t* is
  32 $$ \sum\limits_{\substack{zu \in E(t)}}{\varphi(t-t_{zu})},  $$
  where *φ* is the time decay function that we set *φ*(*t*−*t*_*zu*_):= exp(−*c*(*t*−*t*_*zu*_)) similarly to temporal Katz centrality.

### Results

As the final and main analysis of the relevance of centrality measures, we compute hourly lists of top centrality nodes and calculate the NDCG@50 against the ground truth. We show two different ways to aggregate hourly NDCG@50 values:

1. For each hour of the day between 1:00 and 24:00, we show averages over the days of the tournament.
2. As a single global value, we average NDCG@50 for all days with all hours between 10:00 and 20:00.

**Fig. 10:**
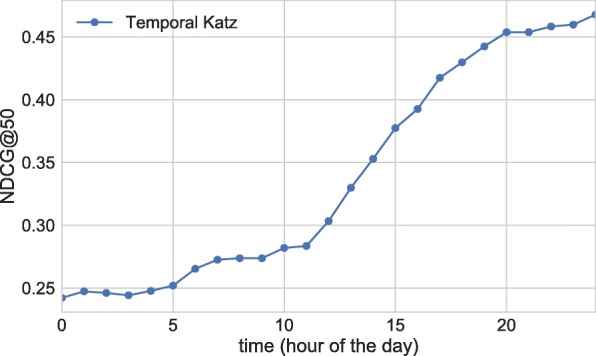
Average daily NDCG@50 performance of temporal Katz centrality on the UO17 data

The hour of the day has a key effect on performance. In the early hours, activity is low, and hence information is scarce to identify the players of the coming day. By contrast, in the late hours after the games are over, we expect that all models easily detect the players of the day based on the tweets of the results. The effect of the hour of the day can be seen in Fig. 10, where we plot the average daily performance for temporal Katz centrality measured over the UO17 data. This observation, along with the fact that daily tennis games start around 10:00 is the motivation to average NDCG@50 scores only between 10:00 and 20:00.

**Fig. 11:**
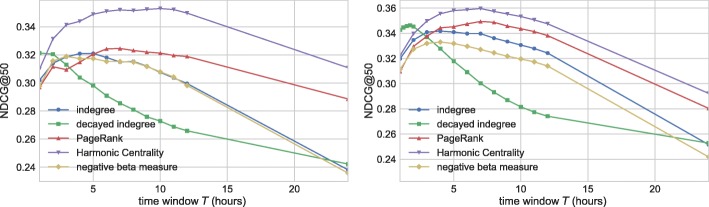
NDCG@50 performance of the baseline methods as the function of time window *T*. For online baseline exponential degree results are shown as the function of half-life *τ*. Left: UO17, Right: RG17

First, we analyze our baseline models. Each static metric is computed at hour *t* over the graph defined by edges arriving in time frame [*t*−*T,t*]. Hence the key parameter of these methods is the length of the time window *T*. Similarly, online decayed indegree depends on the half-life parameter *τ*:= ln2/*c*. Figure 11 shows the overall performance of the static baselines as the function of time frame *T*, and the quality of decayed indegree as the function of half-life *τ*. For both data sets, PageRank and harmonic centrality outperform degree-related methods. Furthermore, these path-based methods prefer larger time frames, while degree-based models perform best at smaller values of *T*.

Next we turn to analyzing temporal Katz centrality with exponential decay. The key parameters of our method are the parameters of the exponential decay *β* and *τ*:= ln2/*c*, and truncation *k*. We then parameterize exponential decay with half-life *τ*:= ln2/*c* instead of *c*.

**Fig. 12:**
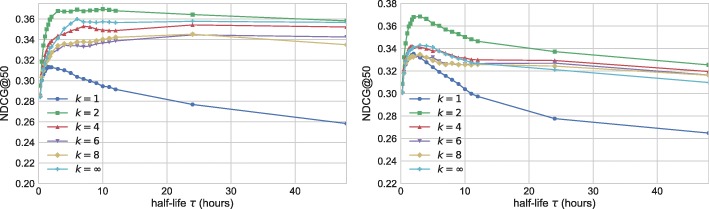
NDCG@50 performance of temporal Katz centrality as the function of half-life parameter *τ*. Different curves correspond to the different values of *k*. We set *β*=1. Left: UO17, Right: RG17

First, we examine the effect of *k* and half-life *τ* by setting *β*=1. Figure 12 shows the performance of temporal Katz centrality at various parameter settings for UO17 the RG17. We plot NDCG@50 against parameter *τ*. Different curves correspond to different *k* parameters. The effect of *k* is significant: Models with *k*>1 strongly outperform models with *k*=1, a very simple version of temporal Katz centrality similar to online degree. The best performance can be achieved on both data sets by setting *k*=2 and *τ*≈3*h*.

**Fig. 13:**
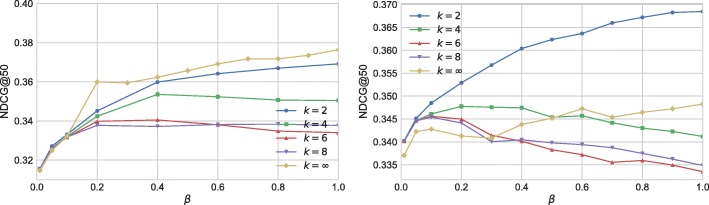
NDCG@50 performance of temporal Katz centrality as the function of parameter *β*. Different curves correspond to the different values of *k*. We set *τ*=6*h* for the UO17 data, and *τ*=3*h* for the RG17 data. Left: UO17, Right: RG17

In Fig. 13 we analyze the importance of parameter *β*. For models with larger *k* (e.g. *k*=8), the importance of *β* is to decrease the effect of paths that are too long, with optimal value around *β*≈0.1−0.2. For methods with lower *k* (e.g. *k*=2), *β* is nearly meaningless, and the use of small *β* in combination with strong exponential decay results in performance deterioration.

**Fig. 14:**
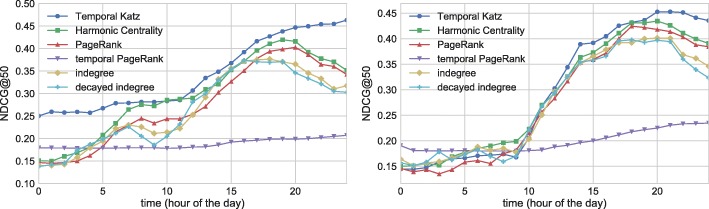
Overall best daily NDCG@50 performance of temporal Katz centrality and the baselines. Left: UO17, Right: RG17

The final conclusion of our experiments is drawn in Fig. 14 where we compare the hourly performance of each method at their best parameter settings. For temporal Katz centrality we set *β*=1,*τ*=3*h,k*=2. In the case of both data sets, temporal Katz centrality can keep up with the performance of harmonic centrality, the strongest baseline model. The quality of temporal PageRank is significantly lower than the quality of other methods. We summarize the best NDCG@50 scores for temporal Katz centrality and the baselines in Table 3. Temporal Katz centrality generally performs better than other baselines. Note that only harmonic centrality, a measure that is static and not online updateable, delivers performance comparable to temporal Katz centrality.

**Table 3:** Best average NDCG@50 performance of each centrality metric

NDCG@50	UO17	RG17
indegree	0.321	0.342
decayed indegree	0.321	0.346
negative beta	0.319	0.333
PageRank	0.325	0.349
temporal PageRank	0.187	0.195
harmonic centrality	0.353	0.359
temporal Katz centrality	0.370	0.368

We illustrate various centrality measures by showing the 20 accounts with highest score for the Roland-Garros semifinals. On June 9, more than 70 players participated in several categories (Men’s singles, Girl’s and Boy’s singles, etc.). In Table 4, we show top accounts at 12:00 by temporal Katz centrality with *k*=*∞* and *τ*=3*h*, and in Table 5 for harmonic centrality and decayed indegree, the latter also at 12:00.

**Table 4:** Temporal Katz centrality with *β*=1.0 (left) and *β*=0.2 (right) top list for RG17 semi final day (June 9) at 12:00

Relevant daily players are highlighted orange. Accounts of players who did not play on this day are highlighted yellow

**Table 5:** Harmonic centrality (left) and decayed indegree (right) top list for RG17 semi final day (June 9) at 12:00

Relevant daily players are highlighted orange. Accounts of players who did not play on this day are highlighted yellow

We show the accounts of tennis players playing participating in the June 9 semifinals in orange and of those who did not play in yellow, for example, women semi-finalists of the previous day, Simona Halep, Timea Bacsinszky, Caroline Garcia and Gabriela Dabrowski. All methods listed 4–6 daily players among the most central 20 accounts. All methods assigned high centrality to Men semi-finalists Rafael Nadal, Andy Murray, Stanislas Wawrinka and Dominic Thiem. Furthermore, temporal Katz centrality with *β*=1.0 and harmonic centrality could recover two additional young daily players, Whitney Osuigwe and Nicola Kuhn. Retired tennis legends Ana Ivanovic and Gustavo Kuerten are not relevant in our experiment as they did not participate in this event.

Notice that decayed indegree and temporal Katz centrality with *β*=0.2 rank sports media accounts (Tennis Channel, WTA, ATP World Tour, Eurosport) higher compared to harmonic centrality and temporal Katz centrality with *β*=1.0. We did not attempt to curate the relevance to media sources, as the number of such Twitter accounts is abundant. Finally, sponsors ‘yonex.com’ and ‘NikeCourt’, as well as the official Twitter account of the event ‘@rolandgarros’ also rank high. Most of these accounts are active every day, with little observable change in time, which justifies why we do not consider them relevant for the temporal evaluation.

## Conclusion

In this paper, we designed an online updateable, dynamic graph centrality measure based on the Katz index. Our proposed metric can incorporate arbitrary time decay functions to emphasize the time-related relevance of the edges based on their time of creation. Our algorithm models information spreading over the stream of edges created subsequently in time.

We presented multiple unsupervised experiments to show that our method can adapt to changes in the distribution of the edge stream. Furthermore, with time decay parameter *c* and *β* we can properly control the effect of recently added edges. We also proved that our metric converges to the Katz index in case of static edge distribution.

In order to assess the quality of our centrality measure, we compiled a supervised evaluation for the mention graphs of Twitter tennis tournament collections along with temporal importance ground truth information. To the best of our knowledge, these are the first Twitter collections enhanced with dynamic node importance labels. We made our data set, as well as our codes publicly available^3^. In our final experiment, we compared our temporal Katz centrality metric with static graph-based measures as well as with other dynamically updateable algorithms. We found that temporal Katz centrality can identify accurately and quickly the emerging, new important nodes and that it worked particularly well in the US Open 2017 (UO17) collection.

## Abbreviations

DCG: Discounted cumulative gain
NDCG: Normalized discounted cumulative gain
RG17: Our twitter data set about Roland-Garros 2017, the French open tennis tournament
TREC: Text retrieval conference
UO17: Our twitter data set about US open 2017, the United States open tennis tournament

## Footnotes

1
https://networkx.github.io/documentation/networkx-1.9.1/reference/generated/networkx.generators. random_graphs.barabasi_albert_graph.html

2 GitHub repository of the temporal PageRank research: https://github.com/polinapolina/temporal-pagerank

3 GitHub repository of our research: https://github.com/ferencberes/online-centrality
